# National health insurance contribution to family planning program funding in Indonesia: A fund flow analysis

**DOI:** 10.12688/gatesopenres.14642.2

**Published:** 2024-01-18

**Authors:** Amirah Ellyza Wahdi, Edward Sutanto, Althaf Setyawan, Yufan Putri Astrini, Nadhila Adani, Halimah Mardani, Nirwan Maulana, Anooj Pattnaik, Trihono Trihono, Siswanto Agus Wilopo

**Affiliations:** 1 Department of Biostatistics, Epidemiology, and Population Health, Faculty of Medicine, Public Health & Nursing, Universitas Gadjah Mada, Sleman, Special Region of Yogyakarta, 55281, Indonesia; 2 Center for Reproductive Health, Universitas Gadjah Mada, Sleman, Special Region of Yogyakarta, 55281, Indonesia; 3 ThinkWell, Central Jakarta, Jakarta Special Capital District, 10350, Indonesia; 4 ThinkWell, 1519 York Road, Lutherville, Maryland, 21093, USA

**Keywords:** Family planning, national health insurance, health financing, Indonesia, universal health coverage, health budget, out-of-pocket payment, private facilities

## Abstract

**Background:**

Launched in 2014, Indonesia’s national health insurance system (JKN) aimed to provide universal health coverage, including contraceptive services, to its population. We aim to evaluate the contribution of JKN to the overall spending for the family planning program in Indonesia.

**Methods:**

Data from the Indonesian Demographic Health Survey, Survey on Financial Flows for Family Planning, Indonesia Motion Tracker Matrix, World Population Prospect, and Indonesian ministries’ budget accountability reports were entered into the CastCost Contraceptive Projection Tool to define budgetary allocation and spending for the family planning program at the national level in 2019.

**Results:**

Indonesia’s family planning program in 2019 was financed mostly by the national budget (64.0%) and out-of-pocket payments (34.6%). There were three main ministries responsible for family planning financing: the National Population and Family Planning Board (BKKBN) (35.8%), the Ministry of Finance (26.2%), and the Ministry of Health (2.0%). Overall, JKN contributed less than 0.4% of the funding for family planning services in Indonesia in 2019. The majority of family planning spending was by public facilities (57.3%) as opposed to private facilities (28.6%).

**Conclusion:**

JKN’s contribution to funding Indonesia’s family planning programs in 2019 was low and highlights a huge opportunity to expand these contributions. A coordinated effort should be conducted to identify possible opportunities to realign BKKBN and JKN roles in the family planning programs and lift barriers to accessing family planning services in public and private facilities. This includes a concerted effort to improve integration of private family planning providers into the JKN program.

## Introduction

In 2014, the Indonesian government launched *Jaminan Kesehatan Nasional* (JKN), a comprehensive national health insurance scheme, with the aim to provide universal health coverage (UHC) to its citizens. Deemed the largest single-payer scheme in the world, JKN, which is financed both by its member’s premium and government subsidy, has covered almost 85% (229.5 million) of all Indonesian citizens by late 2021 ^1, 2^. In addition to providing financial risk protection, JKN, which is managed by the Social Security Administering Body for Health – a separate ministerial-level agency from the Ministry of Health, aims to reduce health inequity and improve service access through reducing regressive payments, such as out-of-pocket (OOP) spending ^1^. Several publications have reported that the implementation of JKN has significantly decreased household OOP spending when accessing health care ^3– 5^, including for family planning (FP) services.

In addition to JKN, the Indonesian health system is characterized by two key features. The first is decentralization, which grants autonomy for district government to manage their health planning, financing, and healthcare services according to local needs ^6^. The second feature is that healthcare services are delivered across both private and public primary health care (PHC) facilities and hospitals ^7^. All public PHC facilities and hospitals are automatically contracted by JKN, but private health facilities need to be contracted by JKN before JKN members can access it without incurring OOP payments. Private midwives are the backbone of FP services in Indonesia, providing 41% of FP services. Yet, only 3% of private midwives received direct reimbursement from JKN by December 2021 ^8^.

A lower-middle income country in Southeast Asia, Indonesia was previously seen as a global FP success story after it halved its total fertility rate (TFR) from 4.9 in 1976 to 2.5 in 2002 ^9^. Yet, Indonesia’s FP progress has stagnated in recent decades as TFR remained the same and the modern contraceptive prevalence rate (mCPR) rate decreased slightly from 57.9% in 2012 to 57.1% in 2017 among married women ^10^. This stagnation also coincides with the decentralization of the Indonesian health system in 2004, in which the influence of national agencies, including the National Population and Family Planning Board, *Badan Kependudukan dan Keluarga Berencana Nasional* (BKKBN), lessened compared to that of local agencies ^11^.

While there are several ministries and ministry-level agencies within the Indonesian government that contribute to FP efforts, BKKBN is the primary agency that is responsible for implementing FP programmes in Indonesia. BKKBN is responsible for the procurement of FP commodities and related consumables in Indonesia, which are then distributed to lower administrative levels. In addition to FP program, BKKBN also implemented reproductive health and family welfare programs, which formed three pillars of population control ^12^. These three pillars must be considered when accounting for the financing for the FP program in Indonesia. However, BKKBN is not under the Ministry of Health and thus, its budget is separate. Other agencies also play significant roles, including the Ministry of Finance which finances FP separately and directly through two funding schemes called the Special Allocation Fund ( *Dana Alokasi Khusus*) and the Family Planning Operational Fund ( *Bantuan Operasional Keluarga Berencana*), while the Ministry of Health publishes clinical guidelines for FP services, and the Coordinating Ministry of Human Development and Cultural Affairs coordinates to ensure alignment in the overall FP program implementation in Indonesia.

Given that JKN streamlined the previously fragmented health insurance system and districts now often have different priority in their FP programs implementation ^13^, JKN was anticipated to further accelerate gains in FP as it includes comprehensive FP services in its benefit service package. In addition to being included as one of the targets in Sustainable Development Goals (SDGs) the inclusion of FP services within benefit service packages of national health insurance schemes have shown to be a cost-saving investment (i.e., prevention of more expensive complications) and results in positive health outcomes (i.e., reduction in unsafe abortions and maternal deaths) ^14– 16^. Individuals may access FP services at all public service delivery points or at private service delivery points that partner with JKN who is then responsible for reimbursing for FP service fees. Yet, studies have shown mixed findings; while recognizing certain subgroups who benefit from the scheme, such as poor individuals or those who use long-acting contraceptives, the implementation of JKN did not increase mCPR generally ^10, 11^. Several systematic reviews have shown that health insurance increases utilization of health services and improves health outcomes both in developed and developing countries ^17– 19^. Yet, existing evidence of the benefit of health insurance and funding specifically for family planning services remains limited.

In order to improve efficiency in funding FP in Indonesia as well as to address its stagnation in TFR and mCPR, it is important to assess the extent of JKN’s contribution to FP funding, recognize funding duplication, and identify any funding gaps in FP programming. Financing for FP is mostly ring-fenced in the national budget and flows from different national ministries, including BKKBN, the Ministry of Health, or directly from the Ministry of Finance to subnational governments (i.e., district BKKBN and local district health offices). Yet, no study has mapped out how funds for FP flow from the national level to the provider level and the contribution JKN makes to this. Thus, we aimed to examine JKN contributions to FP program funding in Indonesia. Findings from this study offer insight into Indonesia’s experience with integrating FP programming into JKN which acts as evidence for other countries aiming to increase their FP indicator performance. Findings from this study may also yield information on financing and regulatory gaps to improve the design of an FP benefit package within a national health insurance scheme.

## Methods

### Data source

This paper used secondary data to construct the FP program fund flow for the fiscal year of 2019, including data from the ministries’ budget accountability reports, the Indonesia Demography and Health Survey (IDHS) ^20^, the Survey on Financial Flows for Family Planning (RFIS), and the Motion Tracker: FP2020 Commitments Activity Report ^21^. For our analysis, we define FP program as aiming to manage childbirth, the ideal age and spacing of childbirth, manage pregnancy, through promotion, protection, and assistance in accordance with reproductive rights utilizing modern contraceptive methods (i.e., condoms, pills, injectables, implants, intrauterine devices [IUDs], tubal ligation, and male sterilization) ^22^. This includes budget line items needed to provide for FP services, such as commodity, personnel, program, and infrastructure. The 2019 World Population Prospect was used to calculate numbers of family planning users ^23 ^. The year 2019 was deliberately chosen to avoid bias due to the coronavirus pandemic that started in March 2020 which shifted funding allocation for programs ^24^. The CastCost Contraceptive Projection Tool developed by the Centers for Disease Control and Prevention (CDC) was used to produce the family planning spending data ^25^. Briefly, the tool is user-friendly spreadsheet, which utilize data from Reproductive Health Surveys or Demographic and Health Surveys, designed to assist countries to estimate quantity of contraceptives' demand and need. A detailed description of the tool is given elsewhere ^26^.

### Data analysis

Budget allocation for the 2019 fiscal year was abstracted from BKKBN, RFIS, the Motion Tracker, and the ministries’ budget accountability reports. These reports provided information on the national budget for FP programming, its distribution through ministries and national agencies and further distribution to the lower administrative levels, as well as foreign donor and non-governmental organization (NGOs) contributions to FP programs in Indonesia.

For spending data, we first interpolated the population of Indonesia in 2019 using data from the 2019 World Population Prospect and the 2017 IDHS ^20, 23^. This calculation resulted in the number of women of reproductive age (WRA), proportion of WRA, and the annual rate of population increase.

We interpolated the projected mCPR in 2019 based on IDHS data from 1997, 2002, 2007, and 2017. The mCPR for each contraceptive method was generated using the same method. All data analysis was conducted using STATA 17. Additional information for sources of contraceptive supplies for each method in 2019 was estimated based on the percentage of current users of each method from 2017 IDHS (Table S1, Extended Data). These numbers were then used for CastCost calculation.

The unit cost for each FP method in the public sector was obtained based on the JKN reimbursement rate (for pills, condoms, IUDs, and implants) and the Indonesia Case Base Groups (for female and male sterilization). The unit cost for FP methods in the private sector was obtained through consultation with the Indonesia Midwives Association Yogyakarta Chapter. The couple-years of protection (CYP) conversion factor was obtained from USAID (see Table S2, *Extended data*) ^27, 28^. Further detailed steps for data analysis can be seen from Table 1.

**Table 1.** Detailed steps taken to simulate the fund flow for family planning services in Indonesia, fiscal year 2019.

Step	Data Used	Description	Output
**1**	• Official data from BKKBN for fiscal year 2019 • Selected national reports on family planning funding	Sorting the data needed to simulate the fund flow based on the origin of the data	• State budget for family planning • Fund distribution through the ministries and agencies • Information on fund flow from the central government to the lower administrative levels
**2**	• World Population Prospect 2019 • 2017 IDHS	Projecting Indonesia population, particularly women of reproductive age, using interpolation	Table 2: • Number of women of reproductive age (WRA), • Annual rate of population increase, • % WRA in a union, and number of WRA in union
**3**	1997, 2002, 2007, 2012, and 2017 IDHS	Applying interpolation to project Indonesia mCPR for fiscal year 2019	Supplementary Table 1: CPR for 2019 for each method and mCPR for fiscal year 2019
**4**	2017 IDHS		Supplementary Table 1: Service distribution for family planning in Indonesia (public vs private)
**5**	• Indonesian Case Base Groups (INA-CBGs) • Stakeholder consultancy • Couple-Years of Protection (CYP)	Inputting the unit cost and CYP for each family planning method to calculate the spending for public and private sector using CastCost	Supplementary Table 2: Family planning spending for public and private sector
**6**	Output of step 1 and Supplementary Table 2	Creating matrices based on the result of Supplementary Table 1	Table 3
**7**	Table 3	Inputting the information to SankeyMATIC	Figure 1

### Data visualization

An Excel spreadsheet was used to map the fund flow and create a family planning fund flow matrix. This paper used SankeyMATIC ( https://sankeymatic.com/) to create a Sankey diagram to visualize the flow of funds.

## Results

### Indonesia demographic background in 2019

In accordance with current regulation in Indonesia, through BKKBN and JKN, the government is responsible for providing a free FP program for all married couples. Based on our analysis, it was interpolated that Indonesia had 72,783,702 WRA in 2019 ( Table 2). This calculation was produced assuming that the annual rate of population increase was 1.06%. Considering that Indonesia’s law stipulated that the FP program was intended for married couples, this calculation was based on the estimation that there were 52,331,482 married women in Indonesia in 2019. Using the IDHS data, the mCPR in 2019 was estimated at 64.2%, with injectables as the most common modern method used by married women in Indonesia.

**Table 2.** Projection of contraceptive needs in Indonesia in 2019 based on IDHS and UN World Population Prospect.

	Year of estimation	
	2017	2019
WRA Age 15-49	72,021,000	72,783,702.4
Annual Rate of Population Increase (%)	1.06	
% WRA in Union	71.90	
Number of WRA in Union	51,783,099	52,331,482
Prevalence by Method (%)		
Tubal ligation	3.8	3.8
Pills	12.1	11.9
IUD	4.7	4.0
Injectable	29	30.9
Condoms	2.5	2.6
Implant	4.7	4.2
Male Sterilization	0.2	0.2
Other modern methods	6.6	6.7
Contraceptive Prevalence Rate: Modern Methods (%)	63.6	64.2

Abbreviation: IDHS, Indonesian Demographic Health Survey; IUD, Intrauterine Device; WRA: Woman of Reproductive Age.

### Public Sector FP Program

**Figure 1.**
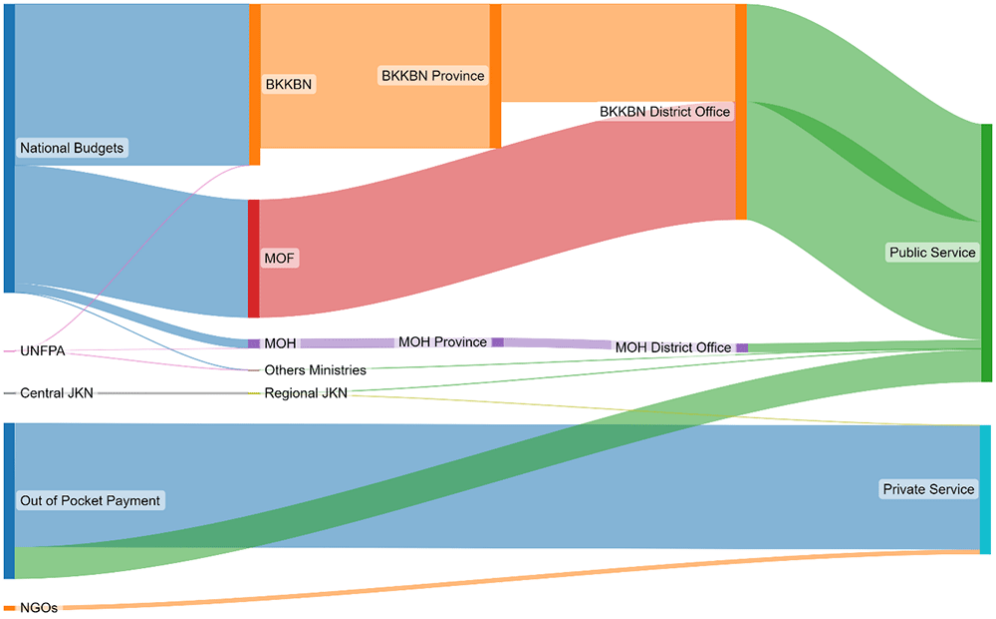
Indonesia family planning fund flow for 2019. Size of each flow corresponds to the overall share of all FP expenditures. Abbreviations: BKKBN, National Population and Family Planning Board; JKN: National Health Insurance; MOF: Ministry of Finance; MOH: Ministry of Health; UNFPA: United Nations Population Fund; NGOs: Non-governmental organizations.

Table 3 and Figure 1 shows that there are three main ministries responsible for family planning financing in Indonesia: the BKKBN (35.8%), the Ministry of Finance (26.2%), and the Ministry of Health (2.0%); thus, in 2019, Indonesia’s FP program was supported mostly by the national budget (64.0%). Furthermore, the majority of family planning spending was at public facilities (57.3%) compared to private facilities (28.6%).

**Table 3.** Family planning fund flow matrix (in USD thousands) for 2019.

Institution(s)	National Budget/ Original Source [%]	Provincial-Level Budget [%]	District-Level Budget [%]	Estimated Expenditure for Public Sector [%]	Estimated Expenditure for Private Sector [%]
BKKBN	252,736 [35.76%]	225,967 [94.10%]	153,009 [43.11%]	153,009 [35.51%]	0 [0.00%]
Ministry of Health	14,160 [2.00%]	14,160 [5.90%]	14,160 [3.90%]	14,160 [3.29%]	0 [0.00%]
Ministry of Finance	185,111 [26.19%]	- *	185,111 [52.16%]	185,111 [42.96%]	0 [0.00%]
Other ministries	258 [0.00%]	- *	- *	258 [0.00%]	0 [0.00%]
JKN †	2,616 [0.37%]	- *	2,616 [0.73%]	28,198 [6.54%]	602 [0.30%]
UNFPA † ‡	163 [0.00%]	- *	- *	163 [0.00%]	0 [0.00%]
Other NGOs †	7,259 [1.02%]	- *	- *	0 [0.00%]]	7,260 [3.59%]
Out-of-pocket Payment †	244,413 [34.58%]	- *	- *	49,988 [11.60%]	194,425 [96.11%]
Total	706,716 [100.00%]	240,127 [100.00%]	354,896 [100.00%]	430,887 [100.00%]	202,287 [100.00%]

Abbreviations: BKKBN, National Population and Family Planning Board; JKN, National Health Insurance; UNFPA: United Nations Population Fund; US$, US Dollar (1 US Dollar is approximately 14,000 Indonesian Rupiah in 2019); NGOs: Non-governmental organizations. *No fund was channelled through the specific level †Institution was not funded through national budget ‡For UNFPA, funding was distributed to BKKBN (US$56,071), the Ministry of Health (US$71,429), and the other ministries (US$35,714).

Around 86% of the total BKKBN budget was allocated for procurement, with 78% budget realization by the end of the fiscal year. Additionally, the BKKBN budget from the national to subnational level was shrinking due to portion of the allocated budget was not executed, cost to that may be utilized to conduct day-to-day operation of the BKKBN offices, and other cost that we were not able to capture in our analysis. For the Ministry of Finance, majority of the budget was used to support operationalizing FP programs (i.e., commodity distribution, personnel transportation cost, and demand generation activity).

In order to simplify the visualization, the JKN contribution for FP programming was included as public sector. The United Nations Population Fund (UNFPA) partnered with the Indonesian government in implementing numerous programs related to FP, therefore UNFPA contribution, unlike that of any other foreign donors and NGOs, was also considered public sector. Service fees for implants, IUDs, tubectomies, and vasectomies are reimbursed within the JKN scheme while other contraceptive methods (e.g., condoms and pills) were not included in this analysis as they are paid through JKN’s capitation payment mechanism regardless of the service rendered. Almost all public sector budget allocation went to public sector health facilities except a small amount of money from JKN that was spent at JKN-contracted private health facilities.

In summary, 64.4% of the overall FP budget in Indonesia, which amounts to US$437 million or 5.66 billion Indonesian Rupiah, was allocated by the public sector in 2019. Around 77.9% of this budget went directly to FP services in the public sector while the remaining budget was allocated for staff salaries at the central and provincial levels.

### Private Sector FP Program

OOP was the main funding source for family planning (34.6% of the overall FP budget in 2019) in the private sector followed by foreign donors and NGOs. Around 80% of all OOP went to the private sector, and the rest went to the public sector. All NGO funds, except those from UNFPA, were spent in the private sector.

### JKN Contribution to FP Program

Our analysis showed that despite having been in effect for five years, JKN contributed less than 0.4% of funding for family planning services in Indonesia in 2019. Around 77% of this spending went to the public sector, while the rest was spent in the private sector.

## Discussion

To the authors’ best knowledge, this is the first study that dives into the details of how FP is funded in Indonesia. This study showed that JKN only contributes a sliver of funding to the provision of comprehensive FP services in Indonesia. Instead, the majority of FP services are funded primarily through the national government budget and OOP. Similar to the result from the Philippines ^29^, JKN’s minimal contribution to overall funding of FP in Indonesia suggests low utilization of the scheme to reimburse FP services. Yet, a previous study in Indonesia that assessed a separate health card program targeting the poor found increased use of contraceptives among females eligible for the program, although this increase was in parallel with an expansion of FP services in public health facilities ^30^. Other studies that have assessed links between insurance status under a UHC scheme and key FP outcomes in other settings found mixed findings. Among the poorest quintile of women in Latin America, insured women had a higher mCPR (16.5%) than uninsured women ^31^. Yet, results from Indonesia, Ghana, and Kyrgyzstan have shown insurance status did not appear to influence mCPR among married women ^10, 32^. These mixed findings may be indicative of the important roles of public versus private FP providers, local sociocultural norms, and the arrangement of FP benefit packages within UHC schemes ^32^. Hence, policymakers should recognize that the inclusion of an FP benefit package to a given UHC scheme alone does not guarantee improved FP outcomes, instead its arrangement should consider various local contexts.

Given BKKBN has contributed in a major way to the provision of FP in Indonesia, there may be a perverse incentive for BPJS to not expand JKN to comprehensively cover FP services. However, given there is still high OOP spending for FP services, especially from private providers who JKN is in a unique position to contract, it may necessitate a rethink of how FP is covered and paid for. While there is no global consensus on the acceptable level of OOP spending, especially for FP context, World Health Organization have defined OOP spending less than 20% of total health expenditures as an indicator of UHC ^33^. The lack of coverage for FP services under JKN is largely due to most women’s preference for private providers like midwives. The majority of FP providers in Indonesia are private sector, with 41% of all FP service provision delivered by private midwives; but approximately only 5% to 36% of private midwives are estimated to be contracted with JKN ^34^. Previous study has showed that this is due to barriers that prohibit private midwives from fully benefiting from the JKN system ^34^. These barriers include inability to directly contract with JKN and suboptimal reimbursement rates ^35^; therefore, efforts to include more private providers under JKN with a better reimbursement system should be a priority.

Our previous qualitative study reported the perception that health care services under the JKN scheme are suboptimal, a preference to skip JKN’s required paperwork or waiting lines, and a preference to access FP services from private providers out of JKN’s network due to proximity as barriers among users ^35^. Additionally, we found that the existence of FP operational assistance funds (government funding separate from JKN funnelled through BKKBN), which can also be used to reimburse FP, is the preferred alternative for private providers in claiming reimbursement for FP services ^35^.

It is interesting to note that our findings show that a portion (20.45%) of OOP in 2019 was spent in the public sector. Ideally, this should not happen as the law in Indonesia guarantees free FP services for all married couples, particularly at public service delivery points. While this study did not explore the clients’ perspective when choosing health facilities at which to obtain FP services, previous studies show that access (e.g., opening hours) and convenience (e.g., waiting time) were major factors in a client’s choice of private service delivery points ^35^. This includes choosing to pay OOP instead of using JKN at public service delivery points. Due to barriers to obtaining FP services using JKN, users may prefer to access public service delivery points as non-JKN patients (i.e., patients who pay OOP or without a referral from lower tier health facilities) instead of using JKN. As all service delivery points, especially hospitals, in Indonesia accept non-JKN patients, this option is seen as a shortcut for wealthier patients.

While Indonesia finances its FP program through several ministries or ministry-level agencies, the majority of FP services in the public sector are funded by the national budget through BKKBN and district BKKBN offices. We could not identify significant additional funding allocated by district governments through the district BKKBN which may be the result of decentralization in Indonesia since 2014. The lower administrative levels have prerogative on how they would like to organize their government, and, in some instances, the BKKBN subdistrict offices would be merged with other offices in line with local government’s various commitments ^36^. As a consequence, the budget for FP programming is often merged with other activities through this institutional integration.

Our study did not find any significant overlap in FP funding, which suggests that the existence of funding duplication is minimal in Indonesia. This shows that there is a clear delineation of each government body’s function and role. Yet, as noted earlier, the existence of separate FP operational assistance funds that have less bureaucratic barriers for subnational units and public providers may contribute to the reduced utilization of JKN ^35^. While such funding may help the provision of FP services on the ground, it is important for policymakers to evaluate both schemes to ensure maximum funding alignment.

The design of this study, which used multiple sources of data to construct the fund flow map, strengthens our estimates for each funding stream. Yet, there are several limitations to this study. First, this study specifically assessed affordability to access FP services, yet there are multiple established factors that also influenced utilization of FP services, including social acceptability, socioeconomic status, and commodity availability, which were not assessed ^37– 39^. Second, while we undertook a massive review to make sure that the data reconciliation could yield the highest quality data, we could not obtain any information from FP commodities manufacturers (e.g., sale and buyer data). Third, this study was not able to provide detailed calculations on spending due to BKKBN’s expanded scope in population control ^12^, which integrated FP programs with reproductive health programs and family welfare programs. Fourth, we were also unable to provide disaggregated funding flows for each modern contraceptive method or line-item budget (i.e., commodity, personnel) in our analysis. Thus, we are unable to dissect each fund flow that was utilized by providers. Further studies should focus on breaking down how the budget is spent on FP to assess efficiency.

## Conclusion

Our study underscores an opportunity to bolster the role of JKN, which currently contributes less than 0.4%, to Indonesia’s FP program, especially as the country renews its pledge to the FP2030 Initiative ^40^. The fact that a significant of FP services are paid for out-of-pocket (34.6%) suggests that barriers still exist for JKN members. To address this, a concerted effort from Government of Indonesia is needed to better align between BKKBN and JKN’s roles in FP programs, and to eliminate barriers to accessing FP services in both public and private facilities. This may involve revising regulations to ensure a high-quality family program is accessible to all Indonesian married couples or integrate private FP service providers into the JKN program.

## Data availability

### Underlying data

Data used in this study are from the household and individual recode dataset of Indonesia in 2017, available from the Demographic and Health Survey (DHS) website. Access to the dataset requires registration and is granted only for legitimate research purposes. A guide for how to apply for dataset access is available at: https://dhsprogram.com/data/Access-Instructions.cfm.

Data was also abstracted from the Survey on Financial Flows for Family Planning (RFIS) (available at extended data link: https://doi.org/10.5281/zenodo.7813127), the Motion Tracker: FP2020 Commitments Activity Report (available at: https://www.motiontracker.org/sites/default/files/documents/TMT%20Activity%20Report%20Indonesia%20%28July%202020%29_Clean%20Version.pdf), and the 2019 World Population Prospect (available at: https://population.un.org/wpp/publications/files/wpp2019_highlights.pdf), and The CastCost Contraceptive Projection Tool (available at: https://archive.cdc.gov/#/details?url=https://www.cdc.gov/reproductivehealth/global/resources-tools/cast-cost/index.htm), thus is considered public domain data.

### Extended data

Zenodo: Extended data for ‘National Health Insurance Contribution to Family Planning Program Funding in Indonesia: A Fund Flow Analysis. https://doi.org/10.5281/zenodo.7813127 ^28^.

This project contains the following extended data:

- Indonesian FP Fund Flow Extended Data 20230410.doc

Data are available under the terms of the Creative Commons Attribution 4.0 International license (CC-BY 4.0).
