# Supplementary material for: National health insurance contribution to family planning program funding in Indonesia: A fund flow analysis
Source: Gates Open Res. 2024 Jan 18;7:105. Originally published 2023 Jul 4. [Version 2] doi: 10.12688/gatesopenres.14642.2 (PMC11006955; doi:10.12688/gatesopenres.14642.2)
Supplement: Peer review history

# Reviewer report: Reviewer response for version 2

**DOI:** 10.21956/gatesopenres.16507.r36118

**Published:** 2024-07-22

**Authors:** Siripen Supakankunti

**Affiliations:** 1 Chulalongkorn University, Bangkok, Bangkok, Thailand

Although the study method had disadvantages, this study addressed them in its limitations. However, limitations can affect the results, leading to conclusions. Another issue to be aware of is insurance. It is not the only factor that affects the effectiveness of FP. Other problems have also been studied, such as educational level, space of children, misconceptions about family planning, and awareness of family planning services that should be identified. Also, family planning educational messages should focus on the involvement of male partners in delivering the service and the benefits of family planning services, as it will help reduce misconceptions about family planning services.

 What should the appropriate TFR rate be in Indonesia? This is important to set the target.

Is the work clearly and accurately presented and does it cite the current literature?

Yes

If applicable, is the statistical analysis and its interpretation appropriate?

Not applicable

Are all the source data underlying the results available to ensure full reproducibility?

Yes

Is the study design appropriate and is the work technically sound?

Partly

Are the conclusions drawn adequately supported by the results?

Partly

Are sufficient details of methods and analysis provided to allow replication by others?

Partly

Reviewer Expertise:

Health Financing, Health Policy, and Strategic Planning, LTC. Health Economics, Economic Evaluation.

I confirm that I have read this submission and believe that I have an appropriate level of expertise to confirm that it is of an acceptable scientific standard, however I have significant reservations, as outlined above.

# Reviewer report: Reviewer response for version 2

**DOI:** 10.21956/gatesopenres.16507.r36122

**Published:** 2024-04-10

**Authors:** Mark Blecher

**Affiliations:** 1 National Treasury of South Africa, Pretoria, South Africa

The report is interesting, useful and concise and highlights the problem of inadequate funding for the family planning programme in Indonesia by the JLN insurance scheme. 

 I suggest a few small improvements:

 i) Table 3 is not completely clear and requires some explanation

 ii) It would be useful to show the unit costs per woman covered 

 iii) The article seems to model the target population as married women...what about unmarried women?

 iv) It would be useful to cite some examples of countries with health insurance systems where the fund is a substantial funder of public health activities such as Thailand.

 -It would be useful if some of the spending trends could have been presented using the classification and relevant axes of the system of National Health Accounts (WHO)

 - Add to my comment in Table 3: For example it is unclear how  row 1 BKKN should be read: How can National budget be $252 m, prov budget $225m, District budget $153m and expenditure $153m. What does this mean and how do these numbers relate to each other?

 -Adding numbers into the useful figure 1 might help to explain the funding flows better

 - Have the authors assessed what programme budget is required and what is the gap? For example through a costing exercise.

 I am in full agreement with the conclusions of the study that JKN needs to support public heath functions such as FP more.

Is the work clearly and accurately presented and does it cite the current literature?

Yes

If applicable, is the statistical analysis and its interpretation appropriate?

Partly

Are all the source data underlying the results available to ensure full reproducibility?

Partly

Is the study design appropriate and is the work technically sound?

Yes

Are the conclusions drawn adequately supported by the results?

Yes

Are sufficient details of methods and analysis provided to allow replication by others?

Partly

Reviewer Expertise:

Health sector financing, public finance, health economics

I confirm that I have read this submission and believe that I have an appropriate level of expertise to confirm that it is of an acceptable scientific standard.

# Reviewer report: Reviewer response for version 2

**DOI:** 10.21956/gatesopenres.16507.r35789

**Published:** 2024-03-05

**Authors:** Darius Erlangga

**Affiliations:** 1 Department of Global Health and Development, London School of Hygiene and Tropical Medicine, London, UK

I have read the revisions, and I am satisfied with the authors’ responses.

Is the work clearly and accurately presented and does it cite the current literature?

Partly

If applicable, is the statistical analysis and its interpretation appropriate?

Not applicable

Are all the source data underlying the results available to ensure full reproducibility?

Yes

Is the study design appropriate and is the work technically sound?

Partly

Are the conclusions drawn adequately supported by the results?

Partly

Are sufficient details of methods and analysis provided to allow replication by others?

Yes

Reviewer Expertise:

Primary health care, health financing, access to care, financial protection, health econometrics

I confirm that I have read this submission and believe that I have an appropriate level of expertise to confirm that it is of an acceptable scientific standard.

# Reviewer report: Reviewer response for version 1

**DOI:** 10.21956/gatesopenres.15960.r34108

**Published:** 2023-08-03

**Authors:** Darius Erlangga

**Affiliations:** 1 Department of Global Health and Development, London School of Hygiene and Tropical Medicine, London, UK

What is the objective of showing Table 3? At the moment, Figure 1 shows a better job of showing the funding flows. Also, It is not clear if there is any link between columns in Table 3, ie any inter-dependence relationship among the three budgets and how you relate between overall budgets and 'estimated expenditure' for the public sector. 

 Ideally, I'd like to see the percentage numbers in Figure 1 to make it more informative. Also, please confirm in the text if the size of the 'flow' corresponds to the overall share of all FP expenditures. Please also indicate what you mean by funding flow. Do you only consider funding intended to pay for health services and commodities? Broadly speaking, funding for health workers to do the services should also be included if you don't indicate otherwise. 

 At the moment, the funding flows figure only shows the journey of each financing source to reach the facility. What would be more interesting is to show how this budget is spent. You might have done it in the text, but it needs to be more systematic, not ad hoc. Given that this is the bulk of the analysis (which is rather very descriptive) you need to spend more time critically appraising the data that you already collected. 

 Is there any reason why the national budget for BKKBN is getting smaller when it flows to the BKKBN District office? To where is the rest of the budget gone? You need to make it clearer

 You mentioned that 86% total BKKBN budget was allocated for procurement. What about the others, e.g. MOF? MOF also allocated a sizeable share of money to Public Service.

 We all need to step back and think about how to interpret these findings. The effect of health insurance on specific health outcomes will depend on whether the insurer intends to cover the related services. BPJS probably already realised that the FP programme has already been covered by BKKBN therefore they see no incentive to expand their funding into the FP programme as well. What's needed is a funding gap analysis to see where the JKN funding could be utilised to strengthen the FP programme without covering the same things as other funders (and avoid perverse incentives among the users and providers)

 What if the BPJS also spend as much as BKKBN to cover the same services? Wouldn't it be considered as double-counting leading to inefficiency? This is why it is important in your analysis to dissect how each funding flow is utilised at facilities. 

 There is a debate in the global community on what is the acceptable level of OOP among patients because realistically it is unrealistic to expect everyone to spend zero given the rise of health burdens and more and more limited resources. Hence the use of catastrophic health expenditure to indicate the level of financial protection. 

 To add to the discussion of how to increase the mCPR, one needs to ask the question what is the biggest barrier to the effective use of FP? Is it really a financial barrier which then could be mitigated by expanding JKN's involvement in covering the said services? or other non-financial barriers, such as acceptability, accommodation, availability, etc.? Access to health services is not determined solely by its affordability but sometimes it can be the trigger. I apologise if the discussion about access might not be your main objective, but yet in the discussion you brought it up so I thought it would be fairer to bring in a broader discussion on access to care.

Is the work clearly and accurately presented and does it cite the current literature?

Partly

If applicable, is the statistical analysis and its interpretation appropriate?

Not applicable

Are all the source data underlying the results available to ensure full reproducibility?

Yes

Is the study design appropriate and is the work technically sound?

Partly

Are the conclusions drawn adequately supported by the results?

Partly

Are sufficient details of methods and analysis provided to allow replication by others?

Yes

Reviewer Expertise:

Primary health care, health financing, access to care, financial protection, health econometrics

I confirm that I have read this submission and believe that I have an appropriate level of expertise to confirm that it is of an acceptable scientific standard, however I have significant reservations, as outlined above.

# Response

**Published:** 2023-11-29

**Authors:** Amirah Wahdi

**Affiliations:** BEPH, Universitas Gadjah Mada, Sleman, Special Region of Yogyakarta, Indonesia

**Point 1: **What is the objective of showing Table 3? At the moment, Figure 1 shows a better job of showing the funding flows. Also, It is not clear if there is any link between columns in Table 3, ie any inter-dependence relationship among the three budgets and how you relate between overall budgets and 'estimated expenditure' for the public sector.

**Response 1:** The objective of showing Table 3 is to ensure that our study findings can be replicated or used for future reference that requires disaggregated numbers, since Figure 1 did not have any numbers to it. We have previously attempted to include USD numbers in Figure 1 directly, but it became more confusing due to the many numbers in Figure 1.

**Point 2: **Ideally, I'd like to see the percentage numbers in Figure 1 to make it more informative. Also, please confirm in the text if the size of the 'flow' corresponds to the overall share of all FP expenditures. Please also indicate what you mean by funding flow. Do you only consider funding intended to pay for health services and commodities? Broadly speaking, funding for health workers to do the services should also be included if you don't indicate otherwise.

**Response 2:** We thanked the Reviewer for this comment. Unfortunately, adding a percentage to each node could lead to a misleading conclusion since the percentage at each node will be the relative

 summation at each node instead of the overall summation of funding flow. However, we agreed on the importance of reporting the proportion for the fund flow and therefore have added the percentage in Table 3.

 We included the definition of the FP program in the Method section to define the FP funding flow further, this includes commodities and personnel costs (funding for health workers to do the service).

**Point 3: **At the moment, the funding flows figure only shows the journey of each financing source to reach the facility. What would be more interesting is to show how this budget is spent. You might have done it in the text, but it needs to be more systematic, not ad hoc. Given that this is the bulk of the analysis (which is rather very descriptive) you need to spend more time critically appraising the data that you already collected.

**Response 3:** We thanked the Reviewer for this suggestion. This study was not intended to explore how the budget was spent since we would need different data sources and methodologies to do that. As we added in the Discussion, that would be the next step from this study, “Further studies should focus on breaking down how the budget is spent on FP to assess efficiency.”. Unfortunately, a limitation of utilizing the Sankey diagram is that we could not illustrate how the budget is spent at each node of the diagram, hence why we include further explanation of how the budget is spent in the text instead.

**Point 4:** Is there any reason why the national budget for BKKBN is getting smaller when it flows to the BKKBN District office? To where is the rest of the budget gone? You need to make it clearer

**Response 4: **We thanked the Reviewer for this question. The reason for the shrinking of the budget from the national level down to the sub-national level is due to multiple factors, which include (1) some of the national FP budgets were never executed and hence did not become an expenditure; (2) the use of the fund to pay the day-to-day operational cost of the BKKBN offices, and (3) other costs that we are not able to capture in this exercise. We have added these details in the Main Text accordingly.  

**Point 5:** You mentioned that 86% total BKKBN budget was allocated for procurement. What about the others, e.g. MOF? MOF also allocated a sizeable share of money to Public Service.

**Response 5: **For the MOF, the majority MOF budget was allocated to provide operational support for the FP program, while MOH and JKN budgets do not majorly purchase commodities. We have added the following sentence: “For MOF, majority of the budget was used to support operationalizing FP programs (i.e., commodity distribution, personnel transportation cost, and demand generation activity).”

**Point 6: **We all need to step back and think about how to interpret these findings. The effect of health insurance on specific health outcomes will depend on whether the insurer intends to cover the related services. BPJS probably already realised that the FP programme has already been covered by BKKBN therefore, they see no incentive to expand their funding into the FP programme as well. What's needed is a funding gap analysis to see where the JKN funding could be utilised to strengthen the FP programme without covering the same things as other funders (and avoid perverse incentives among the users and providers)

**Response 6: **We thanked the Reviewer for this suggestion. We have added the following sentences in the Discussion section to incorporate the Reviewer’s suggestion: “Given BKKBN has contributed in a major way to the provision of FP in Indonesia, there may be a perverse incentive for BPJS not to expand JKN to cover FP services comprehensively. However, given there is still high OOP spending for FP services, especially from private providers who JKN is in a unique position to contract, it may necessitate a rethink of how FP is covered and paid for.”

**Point 7:** What if the BPJS also spend as much as BKKBN to cover the same services? Wouldn't it be considered as double-counting leading to inefficiency? This is why it is important in your analysis to dissect how each funding flow is utilised at facilities.

**Response 7:** As we mentioned in the Introduction, BKKBN and BPJS have different roles in delivering FP programs in Indonesia. BPJS can complement spending on procurement by BKKBN as it is within BPJS's responsibilities to cover the FP service cost (for example, reimbursement to the trained health workers for the services rendered). We do not expect their spending to be equal, but the considerable discrepancies in spending and the high OOP found in our study highlighted a gap in the FP program in Indonesia.

 As noted by the Reviewer, we agreed that if BPJS and BKKBN ended up covering the same services, it would lead to inefficiency. In paragraph #6 of the Discussion section, we have noted the following: “Yet, as noted earlier, the existence of separate FP operational assistance funds that have less bureaucratic barriers for subnational units and public providers may contribute to the reduced utilization of JKN. While such funding may help the provision of FP services on the ground, it is important for policymakers to evaluate both schemes to ensure maximum funding alignment.”

 Unfortunately, our analysis does not allow us to dissect each funding flow utilized at the the provider/facility level. We have added this to the limitation section accordingly.

**Point 8: **There is a debate in the global community on what is the acceptable level of OOP among patients because realistically it is unrealistic to expect everyone to spend zero given the rise of health burdens and more and more limited resources. Hence the use of catastrophic health expenditure to indicate the level of financial protection.

**Response 8:** We thanked the Reviewer for this comment. We have included the following sentences in the Discussion section to accommodate the Reviewer’s comment: “While there is no global consensus on the acceptable level of OOP spending, especially for FP context, World Health Organization have defined OOP spending as less than 20% of total health expenditures as an indicator of UHC.”

**Point 9: **To add to the discussion of how to increase the mCPR, one needs to ask the question what is the biggest barrier to the effective use of FP? Is it really a financial barrier which then could be mitigated by expanding JKN's involvement in covering the said services? or other non-financial barriers, such as acceptability, accommodation, availability, etc.? Access to health services is not determined solely by its affordability but sometimes it can be the trigger. I apologise if the discussion about access might not be your main objective, but yet in the discussion you brought it up so I thought it would be fairer to bring in a broader discussion on access to care.

**Response 9: **We agree with the Reviewer that access to health services, including FP services, is not determined solely by its affordability. We have included the following sentence to provide a more nuanced discussion as suggested by the Reviewer: “First, this study specifically assessed affordability to access FP services, yet there are multiple established factors that also influence the utilization of FP services, including social acceptability, socioeconomic status, and commodity availability, which were not assessed.”

# Reviewer report: Reviewer response for version 1

**DOI:** 10.21956/gatesopenres.15960.r34296

**Published:** 2023-08-02

**Authors:** Lumbwe Chola

**Affiliations:** 1 National Institute of Public Health, Oslo, Norway

**Background**

- Give a good reason or justification for why family planning should be funded through NHI.
- Explain better the roles of the various institutions/agencies. Is BKKBN under the Ministry of Health? If it is under the MOH, why is its budget considered to be separate?
- Include a brief description of the health system in Indonesia. There is need to provide a good picture of the Indonesian context

**Methods**

- Define FP and specify if only looking at modern contraception.
- What methods of contraception are considered? Give the percentage use of contraception methods (I see this was included in the results, but maybe it should be background?)
- Did the authors disaggregate funding for specific contraceptive methods? E.g. are condom campaigns (in HIV program) also considered in this analysis?
- I’d like to know more about CastCost – give a brief description in the main text – could also be great to add details in an annex. What is CastCost and what does it do?
  - And why is it being used for this analysis? The objective seems to state that authors are tracking FP expenditure, not estimating the costs of FP. Or did you set out to do the latter?
- Would be great to look at trends in FP financing before and after introduction of JKN if data is available.
- Also in relation to the above, what has been the overall trend in modern contraceptive use especially in the years before and after JKN.
- Make a distinction between cost, budget and expenditure – these seem to be used interchangeably.
  - Further, justify why budget allocations are being used to track FP funding.
- The authors have not given a clear enough picture of FP funding flows (or of health financing and funding in the overall health system) – the funding flow chart is not well explained and the reader is left with many questions.
  - First, how is the national budget allocated? Is this in a decentralized system? If funding is allocated to FP, is it ring-fenced or is it pooled with funding for other services? If the latter, how was FP funding identified?
- What specific line items are being considered – is it only commodities, or is expenditure on personnel and infrastructure included?
- How is the national health insurance agency financed? Is it independent of the MOH?
- Why is the Ministry of Finance included as a separate FP funder? Does MOF not give money to MOH? If the MOF funding is separate from MOH, please explain what sort of FP programs are being funded separately.

**Results**

- Authors make this statement in the results section:”there are three main ministries responsible for family planning financing in Indonesia: the BKKBN (35.8%), the Ministry of Finance (26.2%), and the Ministry of Health (2.0%)”. Is BKKBN a ministry? See earlier comment about describing the roles of ministries and agencies.
- The discussion under the heading ‘Public Sector FP Program’ is about budget allocation and not expenditure. See earlier comment about giving clarity on budget vs expenditure.
- Private sector FP program: what is the size of the private sector? What is OOP being spent on, commodities? What are the top commodities?
- It is not clear why JKN funds only 0.4% of FP when  FP it ‘includes comprehensive FP services in its benefit service package’ as stated by the authors.

**Discussion**

- The authors state “Similar to the result from the Philippines ^22^ , JKN’s minimal contribution to overall funding of FP in Indonesia suggests low utilization of the scheme to reimburse FP services.” However, the cited article from the Philippines is on a slightly different topic and does not talk about Insurance contribution to FP funding, but gives the overall context of FP in the Philippines.
- “Yet, a previous study in Indonesia that assessed a separate health card program targeting the poor found increased use of contraceptives among females eligible for the program ^23^ ”. This is not entirely true the cited article actually almost states the opposite. Johar M actually states “the demand reinforcement was paralleled with an expansion of family planning services in the public health facilities…”; i.e. the health card has a minimal effect on service utilization.
- Some text in the discussion could have been given in the background section, e.g.:
  “The majority of FP providers in Indonesia are private sector, with 41% of all FP service provision delivered by private midwives; but approximately only 5% to 36% of private midwives are estimated to be contracted with JKN.”
  This statement really does explain why it should not be a surprise that JKN funds a small portion of FP – because the majority of FP providers are in the private sector. Laying out this context earlier in the manuscript could give the reader a better understanding of the situation.
- In the discussion section, authors mention for the first time that a stakeholder consultation or situational analysis:
  “The stakeholder consultation conducted as a part of this analysis showed that this is due to barriers that prohibit private midwives from fully benefiting from the JKN system26.”
  This should be given in the methods section. Who was consulted and how? Were interviews conducted?
- Similarly, this could be mentioned earlier in the background section: “Our previous qualitative study reported the perception that health care services under the JKN scheme are suboptimal, a preference to skip JKN’s required paperwork or waiting lines, and a preference to access FP services from private providers out of JKN’s network due to proximity as barriers among users ^27^ ”
- The much needed context on health financing and particularly provision of family planning services is given in the discussion section. In my opinion, this should come much earlier with a detailed discussion of the role of BKKBN. Please explain the role of BKKBN in the Indonesian health system, and the relationship between BKKBN, JKN and the private sector. An attempt is made to do this in the discussion section, but this also ends up being a little bit confusing
  “Indonesia finances its FP program through several ministries or ministry-level agencies, such as BKKBN, the Ministry of Finance, the Ministry of Health, the Ministry of Interior, the Coordinating Ministry for Human Development and Cultural Affairs, and the National Development Planning Agency; however, the majority of FP services in the public sector are funded by the national budget through BKKBN and district BKKBN offices through financing from the Ministry of Finance. We could not identify significant additional funding allocated by district governments through the district BKKBN which may be the result of decentralization in Indonesia since 2014. The lower administrative levels have prerogative on how they would like to organize their government, and, in some instances, the BKKBN subdistrict offices would be merged with other offices in line with local government’s various commitments ^28^ . As a consequence, the budget for FP programming is often merged with other activities through this institutional integration.”

**Conclusion**

- The conclusion is not really based on the authors findings, but rather speculative. The recommendations are also not solidly anchored on study results

Is the work clearly and accurately presented and does it cite the current literature?

Partly

If applicable, is the statistical analysis and its interpretation appropriate?

Not applicable

Are all the source data underlying the results available to ensure full reproducibility?

Yes

Is the study design appropriate and is the work technically sound?

Partly

Are the conclusions drawn adequately supported by the results?

No

Are sufficient details of methods and analysis provided to allow replication by others?

Partly

Reviewer Expertise:

Health economics/health financing

I confirm that I have read this submission and believe that I have an appropriate level of expertise to state that I do not consider it to be of an acceptable scientific standard, for reasons outlined above.

# Response

**Published:** 2023-11-29

**Authors:** Amirah Wahdi

**Affiliations:** BEPH, Universitas Gadjah Mada, Sleman, Special Region of Yogyakarta, Indonesia

**Point 1: **Background - Give a good reason or justification for why family planning should be funded through NHI.

**Response 1: **We thanked the Reviewer for this question. We added a sentence in the Introduction to provide the rationale: “In addition to being included as one of the targets in Sustainable Development Goals (SDGs the inclusion of FP services within benefit service packages of national health insurance schemes have shown to be a cost-saving investment (i.e., prevention of more expensive complications) and results in positive health outcomes (i.e., reduction in unsafe abortions and maternal deaths)”

**Point 2:** Background - Explain better the roles of the various institutions/agencies. Is BKKBN under the Ministry of Health? If it is under the MOH, why is its budget considered to be separate?

**Response 2: **BKKBN is not under the MOH, hence their budget is separate. We have further expanded the roles of various institutions/agencies in Indonesia that deal with FP in Indonesia in paragraph 4 of the Background section.

**Point 3: **Background - Include a brief description of the health system in Indonesia. There is a need to provide a good picture of the Indonesian context

**Response 3: **We have added a paragraph that briefly Indonesian health system (paragraph #2) in the Background section with the following elaboration:

 “In addition to JKN, the Indonesian health system is characterized by two key features. The first is decentralization, which grants autonomy for district governments to manage their health planning, financing, and healthcare services according to local needs. The second feature is that healthcare services are delivered across both private and public primary health care (PHC) facilities and hospitals. All public primary health care (PHC) facilities and hospitals are automatically contracted by JKN, however private health facilities need to be contracted by JKN before JKN members can access it without incurring OOP payments. Private midwives are the backbone of FP services in Indonesia, with 41% of FP services provided by private midwives.  Yet only 3% of private midwives received direct reimbursement from JKN by December 2021.”

**Point 4: **Methods - Define FP and specify if only looking at modern contraception.

**Response 4: **The definition of FP has been added in the method section, which also specified that this study is only looking at modern contraception, with the following elaboration: “For our analysis, we define the FP program as aiming to manage pregnancy and childbirth, the ideal age and spacing of childbirth through promotion, protection, and assistance in accordance with reproductive rights, utilizing modern contraceptive methods (i.e., condoms, pills, injectables, implants, intrauterine devices [IUDs], tubal ligation, and male sterilization). This includes whole budget line items needed to provide for FP services, such as commodities, personnel, and infrastructure.”

**Point 5: **Methods - What methods of contraception are considered? Give the percentage use of contraception methods (I see this was included in the results, but maybe it should be background?)

**Response 5: **A list of contraception methods has been provided in response to Point #4. The percentage use of each contraception method was included in the results as these numbers are calculated based on our study analysis. Hence it is part of the results of this study (not based on previous references or background information). However, we have provided Indonesia's overall modern contraceptive rate in the Background.

**Point 6: **Methods - Did the authors disaggregate funding for specific contraceptive methods? E.g., are condom campaigns (in HIV programs) also considered in this analysis?

**Response 6: **We could not disaggregate funding for a specific contraceptive method. We have added this as a limitation of this study with the following sentence: “Fourth, we were also unable to provide a disaggregated funding flow for each modern contraceptive methods or line-item budget (i.e. commodity, personnel) in our analysis,....”. Condom campaigns specific to the HIV program were included in this analysis since BKKBN is also responsible for the HIV prevention program through its ‘dual protection program.’ 

**Point 7: **Methods - I’d like to know more about CastCost – give a brief description in the main text – it could also be great to add details in an annex. What is CastCost, and what does it do? And why is it being used for this analysis? The objective seems to state that authors are tracking FP expenditure, not estimating the costs of FP. Or did you set out to do the latter?

**Response 7: **We have added further description about the CastCost tool in the Methods section as requested by the Reviewer: “Briefly, the tool is a user-friendly spreadsheet, which utilizes data from Reproductive Health Surveys or Demographic and Health Surveys, designed to assist countries in estimating the quantity of contraceptives' demand and need.”

 More complete details about the CastCost tool are available directly on the CDC website, which we have referenced in the Main Text. Our objective is to track estimated FP expenditure. However, for the estimated FP expenditure to be tracked/calculated, CastCost required the cost of each contraceptive method to be inputted into the tool.

**Point 8: **Methods - ****It would be great to look at trends in FP financing before and after introduction of JKN if data is available.

**Response 8: **As we noted in the Discussion section, our study is the first to assess FP financing after introducing JKN comprehensively. Hence we are unaware of any other study that compares FP financing before and after JKN introduction.

 However, we have referenced publications that examined the impact of JKN introduction to FP outcomes in the Introduction and Discussion sections, which found national health insurance’s status, while it may reduce out-of-pocket for accessing FP services, did not appear to influence mCPR among married women (Teplitskaya, 2018; Nasution, 2019).

**Point 9: **Methods - Also in relation to the above, what has been the overall trend in modern contraceptive use especially in the years before and after JKN.

**Response 9: **We thanked the Reviewer for this question. The overall trend for mCPR use has been relatively stagnant before and after JKN implementation (in 2014). We have elaborated on this in the Introduction section: “….the modern contraceptive prevalence rate (mCPR) rate decreased slightly from 57.9% in 2012 to 57.1% in 2017 among married women.”

**Point 10: **Methods - Make a distinction between cost, budget and expenditure – these seem to be used interchangeably. Further, justify why budget allocations are being used to track FP funding.

**Response 10: **We thanked the Reviewer for this question. These three terms each refer to a different definition and therefore are not used interchangeably but have been used in accordance with the context.

- Cost: We used “(unit) cost” to refer amount of Rupiah/USD that is incurred to provide each modern contraceptive method to FP acceptors. This cost is required to be inputted into CastCost to allow tracking for FP funding services.
- Budget: We used “budget” to describe the amount of money that the government of Indonesia allocates for FP funding. To address the Reviewer’s question, budget allocation is needed so our FP tracking funding analysis can provide a more complete picture (i.e., an end-to-end examination of how much budget is allocated for FP services to how much estimated funding is fully realized for FPs).
- Expenditure: We used “estimated expenditure” to describe the amount of money realized for FPs in the fiscal year 2019.  

**Point 11: **Methods - The authors have not given a clear enough picture of FP funding flows (or of health financing and funding in the overall health system) – the funding flow chart is not well explained and the reader is left with many questions. First, how is the national budget allocated? Is this in a decentralized system? If funding is allocated to FP, is it ring-fenced or is it pooled with funding for other services? If the latter, how was FP funding identified?

**Response 11: As mentioned in the Introduction section, Indonesia has a decentralized system**. Funding for FP services is mostly ring-fenced. We have included the following elaboration to describe general FP funding flows in Indonesia: “Financing for FP is mostly ring-fenced in the national budget and flows from different national ministries, including BKKBN, the Ministry of Health, or directly from the Ministry of Finance to subnational governments (i.e., district BKKBN and local district health offices)”

**Point 12: **Methods - What specific line items are being considered – Are it only commodities, or is expenditure on personnel and infrastructure included?

**Response 12: **It includes commodities, personnel, and infrastructure to provide FP services. We further specified it in the Methods section: “This includes budget line items needed to provide for FP services, such as commodities, personnel, and infrastructure.”  

**Point 13: **Methods - How is the national health insurance agency financed? Is it independent of the MOH?

**Response 13: **The national health insurance agency is financed by premiums and government subsidies. It is independent of the MOH.

 We have included the following elaboration in the Introduction section: “Deemed the largest single-payer scheme in the world, JKN, which is financed both by its member’s premium and government subsidy, has covered almost 85% (229.5 million) of all Indonesian citizens by late 2021. In addition to providing financial risk protection, JKN, which is managed by the Social Security Administering Body for Health – a separate ministerial-level agency from the Ministry of Health, aims to reduce health inequity and improve service access through reducing regressive payments, such as out-of-pocket (OOP) spending.”

**Point 14: **Methods - Why is the Ministry of Finance included as a separate FP funder? Does MOF not give money to MOH? If the MOF funding is separate from MOH, please explain what sort of FP programs are being funded separately.

**Response 14:** The Ministry of Finance is included as a separate FP funder because it also pays directly for public service (instead of through MOH or BKKBN). This is primarily done through two separate funding schemes (called Special Allocation Fund */Dana Alokasi Khusus* and Family Planning Operational Fund */Bantuan Operasional Keluarga Berencana*) that flow directly from MOF through local government.

 Thus, although MOH or BKKBN budgets are funded by the national budget (that is managed by MOF), the MOF budget for FP is a separate budget from the MOH budget (which may pay for infrastructure or personnel in public primary care facilities) or BKKBN budget (who may pay for FP commodities to be distributed to health care facilities).

**Point 15: **Results - Authors make this statement in the results section:”there are three main ministries responsible for family planning financing in Indonesia: the BKKBN (35.8%), the Ministry of Finance (26.2%), and the Ministry of Health (2.0%)”. Is BKKBN a ministry? See the earlier comment about describing the roles of ministries and agencies.

**Response 15: **Yes, as mentioned in Response #2. BKKBN is a ministerial-level governmental institution. We have clarified this in the Introduction section. 

**Point 16: **Results - The discussion under the heading ‘Public Sector FP Program’ is about budget allocation and not expenditure. See earlier comment about giving clarity on budget vs expenditure.

**Response 16: **As mentioned in Response #10, both terms refer to two different definitions. The budget was the amount of money that the government of Indonesia budgeted for FP services, while (estimated) expenditure refers to the estimated amount of money realized to provide the FP services.

**Point 17: **Results - Private sector FP program: what is the size of the private sector? What is OOP being spent on, commodities? What are the top commodities?

**Response 17: **We cannot establish the size of the private sector in our results as this is out of the scope of this paper. However, in the Introduction and Discussion sections, we referenced other studies that found that private providers deliver a significant proportion of FP services. In this study, we assessed how much FP funding went to private facilities (28.6%).

 In the private sector, OOP is being spent on commodities, personnel, and other costs related to PF services. Unfortunately, our analysis disallowed us to disaggregate OOP spending to specific line-item budgets or types of contraceptive methods. Hence we are not able to answer what OOP is mainly spent on. We have added this as a limitation in our study.

**Point 18:** Results - It is not clear why JKN funds only 0.4% of FP when FP it ‘includes comprehensive FP services in its benefit service package’ as stated by the authors.

**Response 18: **We thanked the Reviewer for this comment. These unfortunate findings are explained in the early part of the Discussion section, where we elaborate:

- “…JKN’s minimal contribution to overall funding of FP in Indonesia suggests low utilization of the scheme to reimburse FP services”,
- “the lack of coverage for FP services under JKN is largely due to most women’s preference for private providers like midwives….; but approximately only 5% to 36% of private midwives are estimated to be contracted with JKN”, and
- “perception that health care services under the JKN scheme are suboptimal, a preference to skip JKN’s required paperwork or waiting lines”.

**Point 19: **Discussion - The authors state “Similar to the result from the Philippines ^22^ , JKN’s minimal contribution to overall funding of FP in Indonesia suggests low utilization of the scheme to reimburse FP services.” However, the cited article from the Philippines is on a slightly different topic and does not talk about Insurance contribution to FP funding, but gives the overall context of FP in the Philippines.

**Response 19: **Both countries are similar in the sense that, although FP is included in the benefit package of national health insurance, there is low utilization to use national health insurance to reimburse FP service.

 The following is the excerpt from the cited articles from the Philippines (Page 6) that showed similarity with our findings in Indonesia: “Across the country, consistently low utilization of PhilHealth FP packages has been reported. The DOH noted that this may have risen from differing interpretations in implementing guidelines among patients and providers (DOH 2019). A 2018 study showed that despite having dedicated FP packages, integrating these services can still be challenging due to issues involving unauthorized fees, lack of capacity, and limited political will (Ross, Fagan, and Dutta 2018).”

**Point 20: **Discussion - “Yet, a previous study in Indonesia that assessed a separate health card program targeting the poor found increased use of contraceptives among females eligible for the program ^23^ ”. This is not entirely true the cited article actually almost states the opposite. Johar M actually states, “the demand reinforcement was paralleled with an expansion of family planning services in the public health facilities…”; i.e. the health card has a minimal effect on service utilization.

**Response 20: **We thanked the Reviewer for this information. As noted by Reviewer, the Johar M’s article does point to “in general, households do not exploit the presence of a health card to increase health care utilisation”. However, Johar M also makes further exceptions for contraceptive service utilization, where he noted there is a significant increase. The following two excerpts of Johar M further substantiate this:

- Result section: “The results are unsurprising given that the reinforcement to enroll is heightened by supply expansion in public facilities around 1997. The AER estimates suggest that eligible females take advantage of both health card coverage and the supply expansion to start using contraception. This result is consistent with Jensen (1996), which finds that Indonesian women’s contraceptive behaviour is highly sensitive to the presence of subsidised facilities. The results from the RAND HIE have also suggested that preventative-type services are particularly price-sensitive because preventative-care is a luxury good as opposed to a normal good and is highly substitutable.”
- Conclusion section: “One area where the program has seemingly encouraged increased utilisation is in contraceptive take-up by eligible females in the households. In this case, however, the demand reinforcement was paralleled with an expansion of family planning services in the public health facilities.”

To provide a more accurate description of Johar M study, we have revised the sentence to the following: “Yet, a previous study in Indonesia that assessed a separate health card program targeting the poor found increased use of contraceptives among females eligible for the program, although this increase was in parallel with an expansion of FP services in public health facilities”

**Point 21: **Discussion - Some text in the discussion could have been given in the background section, e.g.: “The majority of FP providers in Indonesia are private sector, with 41% of all FP service provision delivered by private midwives; but approximately only 5% to 36% of private midwives are estimated to be contracted with JKN.” This statement really does explain why it should not be a surprise that JKN funds a small portion of FP – because the majority of FP providers are in the private sector. Laying out this context earlier in the manuscript could give the reader a better understanding of the situation.

**Response 21:** As suggested by the Reviewer, we have added details about private midwives within the paragraph that elaborated on the Indonesian health system (paragraph #2) in the Background section.

**Point 22: **Discussion - In the discussion section, authors mention for the first time that a stakeholder consultation or situational analysis: “The stakeholder consultation conducted as a part of this analysis showed that this is due to barriers that prohibit private midwives from fully benefiting from the JKN system26.”

 This should be given in the methods section. Who was consulted and how? Were interviews conducted?

**Response 22: **The stakeholder consultation or situational analysis referenced a previous (separate) study that we have conducted and is separate from this study. We apologize for this confusion; we have revised the sentence accordingly.

**Point 23: **Discussion - Similarly, this could be mentioned earlier in the background section: “Our previous qualitative study reported the perception that health care services under the JKN scheme are suboptimal, a preference to skip JKN’s required paperwork or waiting lines, and a preference to access FP services from private providers out of JKN’s network due to proximity as barriers among users ^27^ ”

**Response 23: **We believe that this rationale elaborates on our study findings on why JKN's contribution is very small and should be a part of the Discussion section. We believed that including it in the Background would confuse the reader as that would imply we pre-judged that JKN's contribution to FP is small.

**Point 24: **Discussion - The much needed context on health financing and particularly provision of family planning services is given in the discussion section. In my opinion, this should come much earlier with a detailed discussion of the role of BKKBN. Please explain the role of BKKBN in the Indonesian health system, and the relationship between BKKBN, JKN and the private sector. An attempt is made to do this in the discussion section, but this also ends up being a little bit confusing

 “Indonesia finances its FP program through several ministries or ministry-level agencies, such as BKKBN, the Ministry of Finance, the Ministry of Health, the Ministry of Interior, the Coordinating Ministry for Human Development and Cultural Affairs, and the National Development Planning Agency; however, the majority of FP services in the public sector are funded by the national budget through BKKBN and district BKKBN offices through financing from the Ministry of Finance. We could not identify significant additional funding allocated by district governments through the district BKKBN which may be the result of decentralization in Indonesia since 2014. The lower administrative levels have prerogative on how they would like to organize their government, and, in some instances, the BKKBN subdistrict offices would be merged with other offices in line with local government’s various commitments ^28^ . As a consequence, the budget for FP programming is often merged with other activities through this institutional integration.”

**Response 24: **We thanked the Reviewer for this suggestion. We have moved much of the needed context to the Background section. As mentioned in our responses #2 and #3, we also have explained the role of BKKBN in the Indonesian health system, along with other key stakeholders in the Indonesian health system.

**Point 25: **Conclusion - The conclusion is not really based on the authors findings, but rather speculative. The recommendations are also not solidly anchored on study results

**Response 25: **We thanked the Reviewer for this comment. We have revised the Conclusion section to the following:

 “Our study underscores an opportunity to bolster the role of JKN, which currently contributes less than 0.4%, to Indonesia’s FP program, especially as the country renews its pledge to the FP2030 Initiative40. The fact that a significant of FP services are paid for out-of-pocket (34.6%) suggests that barriers still exist for JKN members. To address this, a concerted effort from Government of Indonesia is needed to better align between BKKBN and JKN’s roles in FP programs, and to eliminate barriers to accessing FP services in both public and private facilities. This may involve revising regulations to ensure a high-quality family program is accessible to all Indonesian married couples or integrate private FP service providers into the JKN program.”
